# Editorial: Global excellence in gastroenterology: Europe

**DOI:** 10.3389/fmed.2023.1188047

**Published:** 2023-03-30

**Authors:** Ludovico Abenavoli

**Affiliations:** Department of Health Sciences, University “Magna Graecia”, Catanzaro, Italy

**Keywords:** microbiota, gastroesophageal reflux disease, small intestinal bacterial overgrowth, irritable bowel syndrome, enteric nervous system

The present Research Topic on the *Global Excellence in Gastroenterology: Europe*, include different articles with the aim to highlight the latest advancements in Gastroenterology across the globe, showcasing the academic excellence and high-quality work of internationally recognized scholars. Also, this collection underline the recent advances maded across the area of Gastroenterology and disccuss several pivotal future challenges faced by researchers.

In the first article of this Topic, Gubensek et al. compare the efficacy of both insulin and plasma exchange (PE) to treat hyper-triglyceridemic acute pancreatitis (HTG-AP). A randomized, parallel group study in HTG-AP patients with non-severe prognosis and triglycerides, has been performed. Patients were randomized to daily PE or insulin infusion until triglycerides were <10 mmol/L. Primary outcome was the reduction in triglycerides within 24 h. Secondary outcomes were days needed to reduce triglycerides below 10 mmol/L, highest C-reactive protein CRP level during treatment and percentage of patients with a severe course of pancreatitis. The Authors found no significant difference, but only a trend toward a greater decrease in triglycerides with PE, with a comparable clinical course. These preliminary data do not support the universal use of PE in patients with HTG-AP.

The gut microbiota is a complex ecosystem harboring our intestine. They maintain human body equilibrium, while their derangement, namely, dysbiosis, has been associated with several gastrointestinal diseases, including liver disease. Small intestinal bacterial overgrowth (SIBO) is an example of dysbiosis of the upper gastrointestinal tract. Considering this background, Scarpellini et al. have evaluated the relationship between SIBO, endotoxemia and the grade of liver steatosis and fibrosis in hepathopatic patients. The results shows that SIBO prevalence and relative endotoxin blood levels seem to be significantly associated with the grade of liver fibrosis. Also, a significant increase in SIBO prevalence in cirrhotic patients, has been described.

Gastrointestinal (GI) bleeding is associated with considerable morbidity and mortality. Red blood cell transfusion has long been the cornerstone of treatment for anemia due to GI bleeding. To optimize the management of anemia and iron deficiency in adults with acute or chronic GI bleeding, Montoro et al. developed a new protocol in collaboration with healthcare professionals, including internal medicine physicians, intensive care specialists, and hematologists.

Irritable bowel syndrome (IBS) is a common condition that affects the digestive system and involves 10–23% of adults worldwide. Zhang, Ma et al. analyze the evolution and trends of IBS literature between 2007 and 2022. This bibliometric point of view offers for the first time a deep insight into the current status of this very interesting topic. USA and Sweden are dominant in the IBS field with a high number of publications, great scholarly impact, and broad collaboration network.

Gastroesophageal reflux disease (GERD) affects an estimated 10–30% of the Western population. To evaluate the knowledge structure, evolution of research themes, and emerging topics of GERD, Zhang, Zhang et al. proposes a bibliometric approaches to this subject. In particular the Authors found that current studies in this field are focused on the differential diagnosis of GERD with several conditions.

In the last few years, research has shown the potential benefits of fecal microbiota transplantation (FMT) in IBS subjects. Acute infectious gastroenteritis is a well-established risk factor for developing IBS defined as post-infectious IBS (PI-IBS). Tkach et al. assess FMT's safety, clinical and microbiological efficacy in patients with PI-IBS. The Authors demostrated the effectiveness of FMT use compared to traditional pharmacotherapy in patients with PI-IBS.

Another study by Gentile et al. analyzed through a survey some models of the policies, approaches, and solutions that address the social and health unmet needs of patients with IBS. The Authors identified three main needs to improve IBS patient's lifestyle, and in particular: access to psychological support, supporting diet and adapted physical activity, and home-based digital health support.

The enteric nervous system (ENS) continues to dazzle scientists with its ability to integrate signals, from the outside as well as from the host, to accurately regulate digestive functions. Composed of neurons and enteric glial cells, the ENS, interplays with numerous neighboring cells through the reception and/or the production of several types of mediators. In particular, ENS can produce and release n-6 oxylipins. These lipid mediators derive from arachidonic acid, are involved in inflammatory and allergic processes, and can also regulate the functions of both immune and nervous system. The review by Mantel et al. discuss on the relationship between n-6 oxylipins and digestive functions in the context of ENS.

Gastroenterology is an active discipline and an evolving area within pre-clinical and clinical domains. New therapies, modern techonogical tools and a deep knowledge of the pathogenesis are the main drivers of this growth. In this context, this Research Topic can represent for the scholars a useful and updated point of view to underline the last novelties in the context of global excellence in the field of Gastroenterology.

## Author contributions

LA: conceptualization, original draft preparation, and editing.

